# Robust chirality through merging BICs

**DOI:** 10.1038/s41377-026-02294-6

**Published:** 2026-04-23

**Authors:** Jue Li, Haoye Qin, Qinghua Song

**Affiliations:** 1 Tsinghua Shenzhen International Graduate School, Tsinghua University, Shenzhen, 518055 China; 2 Department of Electrical and Computer Engineering, National University of Singapore, Singapore, 117583 Singapore

**Keywords:** Metamaterials, Photonic crystals, Photonic devices, Nonlinear optics, Applied optics

## Abstract

Robust chirality is demonstrated by exploiting the merging of multiple accidental bound states in the continuum (BICs). This mechanism simultaneously sustains ultrahigh-quality factor *Q* resonances and strong chiroptical responses across a wide region of momentum space, achieving near-perfect circular dichroism (~0.99) and an ultrahigh-*Q* value (~10⁴) in a planar dielectric platform.

Chirality plays a central role in chemistry, biology, and pharmaceutical science, where molecules with opposite handedness can exhibit dramatically different biological activities. Optical methods based on circularly polarized light have long served as an essential tool for distinguishing chiral structures, with circular dichroism (CD) spectroscopy being one of the most widely used techniques^1,2^. However, the intrinsic chiroptical response of natural materials is typically extremely weak, posing a fundamental challenge for sensitive detection and manipulation of chiral light–matter interactions.

In recent years, metasurface arrays of subwavelength nanostructures, capable of tailoring the amplitude, phase, and polarization of light, have emerged as a powerful platform for enhancing chiroptical effects^3–6^. By engineering resonant electromagnetic modes, metasurfaces can significantly amplify CD and optical activity. Among the various approaches, bound states in the continuum (BICs) have attracted particular attention. These radiation-suppressed modes theoretically possess infinite *Q* values and enable extremely strong light-matter interactions when converted into quasi-BIC resonances through controlled symmetry breaking^7–10^.

In 2019, Wenzhe Liu et al.^11^ revealed that BICs can split into a pair of circularly polarized singularities (*C* points) with opposite half-integer topological charges through in-plane inversion symmetry breaking, thereby establishing a fundamental connection between BICs and *C* points. Building on this concept, Adam Overvig et al.^12^ demonstrated ultrasharp Fano resonances based on chiral quasi-BICs using three-dimensional meta-atoms, enabling near-unity CD. Subsequently, Tan Shi et al.^13^ proposed a planar chiral metasurface supporting strong chiroptical responses with a moderate *Q* value (~121) and near-perfect CD (~0.93). However, these previous experimental demonstrations of strong CD relied on false (extrinsic) chirality, achieved through oblique illumination or structural anisotropy. A significant step toward intrinsic chirality was reported in 2023 by Yang Chen et al.^14^, who realized true chiral responses in resonant metasurfaces with a *Q* value of ~2663 by introducing a slanted geometry that simultaneously breaks both in-plane and out-of-plane symmetries. Meanwhile, several approaches have been explored to achieve wide-angle chiral responses. For instance, Minho Choi et al.^15^ employed flat-band engineering to achieve wide-angle chirality, although the performance remained limited (CD ≈ 0.6, *Q* ≈ 140) due to insufficient robustness. Meanwhile, Wenjing Lv et al.^16^ investigated magneto-optical BICs, which can circumvent the need to break geometrical symmetry and theoretically maintain infinite-*Q* values resonances while simultaneously enabling near-unity CD (~1).

Despite these advances, most existing approaches either confine strong CD to isolated *k*-points in momentum space or remain highly sensitive to fabrication imperfections and angular misalignment. In addition, magneto-optical BICs typically require external magnetic fields and specific material platforms, posing substantial challenges for practical photonic integration. These limitations highlight the urgent need for a purely dielectric and fabrication-friendly strategy capable of simultaneously achieving ultrahigh *Q* values and near-perfect CD while maintaining robustness against geometric perturbations and angular dispersion. Recently, a newly published work in *eLight* addressed this challenge by exploiting the merging of multiple accidental BICs, demonstrating robust high-*Q* chiral responses over a wide region in momentum space^17^.

As illustrated in Fig. 1a, the authors introduce a planar dielectric chiral metasurface device and its design workflow for realizing robust ultrahigh-*Q* intrinsic chirality. The metasurface is engineered to support multiple accidental BICs with a net zero topological charge. The underlying physical mechanism is illustrated in Fig. 1b. By lifting a Dirac-type degeneracy through controlled in-plane and out-of-plane symmetry breaking, the system undergoes a deterministic topological evolution in momentum space. During this process, *C* points with identical handedness migrate toward the *Γ* point, while those with opposite handedness annihilate or shift to higher-*k* regions. The resulting clustering of *C* points produces a broad region of strong chiroptical response rather than the isolated singularities typically observed in conventional quasi-BIC metasurfaces. As shown in Fig. 1c, d, this topology-enabled mechanism leads to the robust simultaneous realization of ultrahigh-*Q* resonances and strong chiral responses, manifested across a wide region of the parameter space and momentum space. As a result, the device achieves an ultrahigh-*Q* value approaching 10⁴ together with near-unity CD (~0.99) across a wide momentum (angular) range ( | *k*_*x*_*P*/2π, *k*_*y*_*P*/2π | < 0.06). Moreover, across the parameter space, the metasurface simultaneously maintains a CD above 0.9 and a *Q* value exceeding 5000, highlighting the robustness of the proposed design. Beyond linear responses, the metasurface further supports nearly perfect nonlinear chirality (~0.999) in third-harmonic generation, highlighting the potential of this topology-enabled approach for robust and scalable chiral photonic devices.

**Fig. 1:**
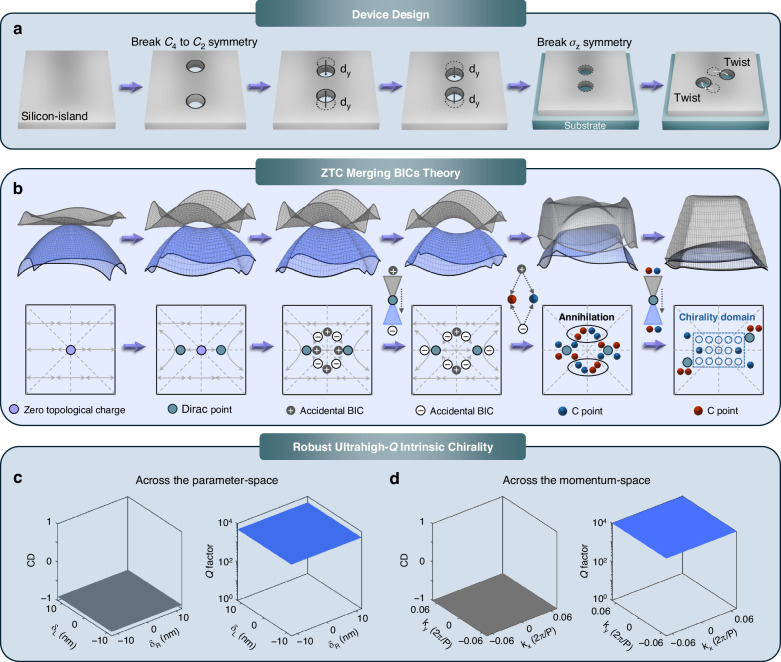
Conceptual illustration of a robust ultrahigh-*Q* intrinsic chiral metasurface device. **a** Schematic rendering of the device architecture and design workflow for realizing robust ultrahigh *Q* values and near-perfect intrinsic chirality. **b** Illustration of the underlying mechanism based on the zero-topological-charge (ZTC)-mediated merging of accidental bound states in the continuum (BICs). Simultaneous realization of robust ultrahigh-*Q* resonances and ultrahigh chiral responses emerging across a wide region of the (**c**) parameter space and (**d**) momentum space

Recently, the merging of multiple BICs has opened new opportunities to achieve ultrahigh-*Q* resonances over an expanded region of momentum space^18–20^. By exploiting the merging of multiple accidental BICs, the present work establishes a robust route toward planar dielectric chiral metasurfaces that simultaneously support near-perfect CD and ultrahigh-*Q* resonances across a wide momentum domain. Importantly, the demonstrated resilience against geometric perturbations and angular dispersion addresses a long-standing challenge in translating chiral metasurfaces from proof-of-concept demonstrations to practical photonic devices. Looking forward, such robust high-*Q* chiral platforms may enable a new generation of compact photonic technologies, including ultrasensitive chiral sensing, nonlinear chiral light sources, and integrated chiral photonic circuits, thereby opening promising opportunities for advanced optical information processing and light-matter interaction engineering.
